# Infections and immune dysregulation in ataxia-telangiectasia children with hyper-IgM and non-hyper-IgM phenotypes: A single-center experience

**DOI:** 10.3389/fped.2022.972952

**Published:** 2022-10-20

**Authors:** Aleksandra Szczawińska-Popłonyk, Katarzyna Tąpolska-Jóźwiak, Eyal Schwartzmann, Barbara Pietrucha

**Affiliations:** ^1^ Department of Pediatric Pneumonology, Allergy and Clinical Immunology, Institute of Pediatrics, Poznań University of Medical Sciences, Poznań, Poland; ^2^ Poznań University of Medical Sciences, Medical Student, Poznań, Poland; ^3^ Department of Immunology, Children’s Memorial Health Institute, Warsaw, Poland

**Keywords:** ataxia-telangiectasia, inborn errors of immunity, syndromic immunodeficiency, infection, immune dysregulation, malignancy

## Abstract

Ataxia-telangiectasia (A-T) is a severe syndromic neurodegenerative inborn error of immunity characterized by DNA reparation defect, chromosomal instability, and hypersensitivity to ionizing radiation, thereby predisposing affected individuals to malignant transformation. While the leading disease symptomatology is associated with progressively debilitating cerebellar ataxia accompanied by central and peripheral nervous system dysfunctions, A-T is a multisystemic disorder manifesting with the heterogeneity of phenotypic features. These include airway and interstitial lung disease, chronic liver disease, endocrine abnormalities, and cutaneous and deep-organ granulomatosis. The impaired thymic T cell production, defective B cell development and antibody production, as well as bone marrow failure, contribute to a combined immunodeficiency predisposing to infectious complications, immune dysregulation, and organ-specific immunopathology, with the A-T hyper-IgM (HIGM) phenotype determining the more severe disease course. This study aimed to clarify the immunodeficiency and associated immune dysregulation as well as organ-specific immunopathology in children with A-T. We also sought to determine whether the hyper-IgM and non-hyper-IgM phenotypes play a discriminatory role and have prognostic significance in anticipating the clinical course and outcome of the disease. We retrospectively reviewed the medical records of twelve A-T patients, aged from two to eighteen years. The patients' infectious history, organ-specific symptomatology, and immunological workup including serum alpha-fetoprotein, immunoglobulin isotypes, IgG subclasses, and lymphocyte compartments were examined. For further comparative analysis, all the subjects were divided into two groups, HIGM A-T and non-HIGM A-T. The clinical evaluation of the study group showed that recurrent respiratory tract infections due to viral and bacterial pathogens and a chronic obstructive airway disease along with impaired humoral immunity, in particular complete IgA deficiency, were noted in all the A-T patients, with both HIGM and non-HIGM phenotypes. The most important features with the discriminatory role between groups, were autoimmune disorders, observable four times more frequently in HIGM than in non-HIGM A-T. Two patients with the HIGM A-T phenotype were deceased due to liver failure and chronic Epstein-Barr virus (EBV) infection. It may therefore be assumed that the HIGM form of A-T is associated with more profound T cell dysfunction, defective immunoglobulin class switching, chronic EBV expansion, and poorer prognosis.

## Introduction

Ataxia-telangiectasia (A-T) is an autosomal recessive syndromic inborn error of immunity (IEI) (1) characterized by the DNA reparation defect, genomic instability, and sensitivity to ionizing radiation with multisystemic involvement and unfavorable outcome. The disease pathophysiology results from mutations in the *Ataxia-Telangiectasia Mutated* (*ATM*) gene encoding for a protein kinase which is a member of the large phosphoinositidil 3-kinase related protein kinase (PIKK) family. The nuclear and cytoplasmic enzymatic ATM kinase activity is pleiotropic and heterogeneous. The enzyme plays an important role in controlling the cell-cycle checkpoints and coordinating the cellular signaling pathways in response to DNA double-strand breaks (DSBs), genotoxic factors, and oxidative stress. It is also involved in cytoplasmic processes, phosphorylating numerous substrates important in mitochondrial respiration and energy metabolism. In A-T, the disruption of the multiplicity of ATM kinase functions in DNA damage response, transcription and translation regulation, protein aggregation, and autophagy, maintaining cellular homeostasis (2–6) is underpinning the multisystemic involvement with the multiplicity of phenotypic features (7–9). The leading A-T symptomatology is characterized by neurodegeneration and progressively debilitating cerebellar ataxia with postural instability, oculomotor apraxia, dysarthria and orolingual insufficiency, as well as extrapyramidal dysfunctions with choreoathetotic movements, dystonia and muscle tremor (4, 10–12). The extended disease phenotype also includes chronic obstructive airway and interstitial lung disease (13–16), chronic inflammatory non-alcoholic liver disease (17–20), cutaneous and systemic granulomatosis (21–24), and hormonal dysfunctions with growth retardation, gonadal insufficiency, and diabetogenic insulin resistance (25–27). The impaired thymic T cell production, defective B cell development accompanied by hypogammaglobulinemia, IgG subclass and antigen-specific antibody generation, and bone marrow failure contribute to a combined immunodeficiency (28–30). The more severe clinical disease course with infectious complications, immune dysregulation, organ-specific immunopathology, and a high risk of lymphoid malignancy has been assigned to the phenotype characterized by low serum IgG and/or IgA and normal to elevated IgM immunoglobulin isotype levels corresponding with the hyper-IgM (HIGM) A-T variant (31–34).

This study aimed to clarify the immunodeficiency and associated infectious complications, immune dysregulation as well as organ-specific immunopathology in children with A-T. We also sought to determine whether the hyper-IgM (HIGM) and non-hyper-IgM (non-HIGM) phenotypes play a discriminatory role and have prognostic significance in anticipating the clinical course of the disease.

## Patients and methods

### The study group

Medical records of twelve A-T children, six boys and six girls, aged from two to eighteen years who have been followed and treated in our tertiary care university department of pediatric immunology, have been reviewed. The clinical analysis comprised the individual patients' infectious history with particular emphasis on respiratory complications, autoimmune disorders, and organ-specific symptomatology. The immunological laboratory work-up included serum immunoglobulin G, M, and A isotypes, IgG subclass levels, and peripheral blood B and T lymph cell flow cytometric immunophenotyping. The alpha-fetoprotein (AFP) activity was also examined in all the children studied.

Referring to the individual patients' IgM levels, further comparative analysis and dividing all the subjects into two groups, HIGM A-T and non-HIGM A-T was done. Herein, we stratified patients as HIGM A-T when their serum IgG and/or IgA levels were decreased with IgM levels above the reference values and non-HIGM A-T when their serum IgM levels were normal or below the reference ranges.

### The peripheral blood lymph cell flow cytometric approach

Peripheral venous blood samples anticoagulated with ethylenediaminetetracetic acid (EDTA-K_2_) were stored at a temperature of between 4 and 8°C and processed within 24 h. Cells were labelled with the following murine fluorochrome-stained monoclonal antibodies: anti-CD45 FITC (fluoresceine isothiocyanate), anti-CD14 PE (phycoerithrin), anti-CD19 PE, anti-CD19 PerCP (peridinin chlorophyll protein), anti-IgM FITC, anti-IgD FITC, anti-CD38 APC (allophycocyanin), anti-CD27 PE, anti-CD21 FITC, as well as anti-CD3 FITC, anti-CD4 FITC, CD45RA FITC, CD127 FITC, CD185 FITC, anti-CD8 PE, anti-CD16 + CD56 PE, CD25 PE, CD31 PE, CD45RO PE, anti-CD3 PerCP, CD197 PerCP, anti-CD4 APC and anti-CD8 APC (all Beckton-Dickinson Biosciences, USA).

Blood samples were mixed with antibodies, incubated in a lysing solution (FACS Lysing Solution, Beckton-Dickinson, USA), centrifuged twice, and suspended in a phosphate buffered saline (PBS, Roche, Germany). The acquisition of cells and analysis were carried out with the use of the flow cytometer FACSCanto and FACSDiva software (Beckton-Dickinson, USA). On biparametric scattering CD45 + CD14- lymphocytes, the following sequential gating strategies for the characterization of lymphocyte subpopulations were applied:

B cells were identified as CD19-expressing cells in the lymphocyte population, and CD19+ B cells were then analyzed either for the expression of CD27 and IgD, or CD21 and CD38 and IgM. The following B cell subsets were delineated: immature CD19 + CD21lo, immature activated CD19 + CD38loCD21lo, transitional CD19 + CD38hisIgMhi, non-switched memory CD19 + CD27 + sIgD+, switched memory CD19 + CD27 + IgD- B cells, and CD19 + CD38hisIgM- plasmablasts.

T cells were identified as CD3-expressing cells in the lymphocyte population and analyzed either for the expression of CD4 and CD8. Subsequently, CD3 + CD4+ T helper cells, and CD3 + CD8+ T cytotoxic cells were delineated.

T helper cells were then analyzed for either the expression of CD27 and CD45RO or CD45RA, or CD31, CD127 and CD25, or CD185. This approach enabled to identify the following T helper cell subsets: CD3 + CD4 + CD31 + CD45RA + recent thymic emigrants, naïve CD3 + CD4 + CD27 + CD45RA+, regulatory CD3 + CD4 + CD25 ++ CD27-, central memory CD3 + CD4 + CD27 + CD45RO+, effector memory CD3 + CD4 + CD27-CD45RO+, terminally differentiated CD3 + CD4 + CD27-CD45RA+, follicular CD3 + CD4 + CD185 + CD45RO+, and regulatory CD3 + CD4 + CD45RO + CD127-CD25++ T helper cells.

Among CD3 + CD8 + cytotoxic T cells, analyzed for the expression of CD197, CD27 and CD45RO or CD45RA, the following subsets were distinguished: naïve CD3 + CD8 + CD197 + CD27 + CD45RA+, central memory CD3 + CD8 + CD197 + CD27 + CD45RO+, effector memory CD3 + CD8 + CD197-CD27-CD45RO+, and terminally differentiated CD3 + CD8 + CD197-CD27-CD45RA + cells.

NK cells were defined as CD3- and CD16 + and/or CD56 + cells.

The relative values of PB lymphocytes, the B, T, and NK cells of the total lymphocyte population as well as B and T cell subsets were calculated. The absolute counts of all cell subsets were calculated from the PB leukocyte counts. A comparative analysis was done with the reference cut-off values of B and T cell subsets for pediatric populations of different age groups.

### Statistical analysis

Due to the small patient sample size, resulting from the rarity of the disease, statistical methods were not employed and a descriptive showcasing of the study group was done.

## Results

### Immunodeficiency

In all A-T affected patients, humoral immunodeficiency was observable. Whereas the elevated IgM levels were reflecting a defective class switch recombination (CSR) process discriminatory for the two HIGM and non-HIGM patient groups, IgA deficiency was revealed in all but one patient in each group. While serum IgG, IgG1 and IgG2 subclass levels were decreased below the reference values in all the HIGM A-T patients, in three out of six non-HIGM patients, IgG and IgG1 subclass levels were within the reference values. In the latter group, in one patient only, a low IgG3 subclass level was shown, whereas IgG4 levels were normal in all six patients. In flow cytometric PB lymphocyte analysis, impaired B and T lymph cell differentiation and maturation were revealed. Persistent lymphopenia, abnormal B cell neogenesis with low B cell numbers, and defective development of memory B cells were observable in all patients. T cell lymphopenia with low numbers of recent thymic emigrants (RTE) reflecting thymic dysfunction in generating naive T cells, with low CD4 + CD45RA: CD4 + CD45RO + ratio alike was the universal feature of both HIGM and non-HIGM A-T phenotypes. In all the A-T patients studied, both HIGM and non-HIGM, serum AFP activity was remarkably elevated (reference serum level <8 ng/ml). The results of the immunological workup in the A-T children studied are shown in Table 1.

**Table 1:** The immunological workup of twelve A-T patients studied with discrimination between HIGM and non-HIGM phenotypes.

Phenotype		HIGM-AT						Non-HIGM-AT					
Patient No		Pt 1	Pt 2	Pt 3	Pt 4	Pt 5	Pt 6	Pt 7	Pt 8	Pt 9	Pt 10	Pt 11	Pt 12
Age [years]		2	4	5	6	9	17	2	3	4	14	18	18
AFP [ng/ml]		63.84 ↑	124.40 ↑	123.50 ↑	182.00 ↑	215.70 ↑	129.70 ↑	71.37 ↑	111.30 ↑	145.20 ↑	395.70 ↑	161.20 ↑	133.25 ↑
Immunoglobulins [mg/dl]	IgG	125 ↓	221 ↓	215 ↓	312 ↓	330 ↓	223 ↓	1116 N	1084 N	374 ↓	418 ↓	363 ↓	805 N
	IgM	1466 ↑	510 ↑	374 ↑	2099 ↑	381 ↑	963 ↑	155 N	58 N	68 N	28 ↓	76 N	48 N
	IgA	3 ↓	1 ↓	0 ↓	68 N	2↓	1 ↓	74 N	2 ↓	7 ↓	2 ↓	35 ↓	23 ↓
IgG subclasses [mg/dl]	IgG1	39.80 ↓	182.20 ↓	135.60 ↓	245.10 ↓	280.80 ↓	147.80 ↓	1110.30 ↑	344.50 N	238.80 ↓	374.80 N	245.00 ↓	460.80 N
	IgG2	41.87 ↓	36.30 ↓	25.40 ↓	55.22 ↓	53.45 ↓	117.10 ↓	82.81 N	27.12 ↓	98.64 N	117.34 N	97.50 N	138.45 N
	IgG3	83.04 ↑	11.86 N	12.43 N	8.99 ↓	3.39 ↓	16.35 ↓	91.02 ↑	56.06 N	69.31 N	34.51 N	25.23 ↓	41.31 N
	IgG4	13.10 N	1.01 ↓	1.41 ↓	3.22 N	7.00 ↓	11.64 ↓	1.68 N	11.20 N	11.67 N	23.91 N	12.55 N	18.10 N
Peripheral blood lymphocytes [cells/%]	Lymph total	3628/44% N	763/24% ↓	1142/32% ↓	2826/26% N	1067/18% ↓	305/20% ↓	701/13% ↓	2165/33% N	939/23% ↓	1235/15% ↓	482/16% ↓	825/21% ↓
	B CD19+	529/50% N	83/10% ↓	108/9% ↓	339/12% N	93/9% ↓	2/1% ↓	122/16% ↓	243/11% ↓	92/10% ↓	66/5% ↓	21/4% ↓	121/14% N
	Naϊve B CD19 + CD27-sIgD+	531/50% N	28/34% ↓	47/44% ↓	379/80% N	44/47% ↓	0 ↓	56/46% ↓	133/55% ↓	73/79% ↓	46/70% ↓	16/76% ↓	102/84% N
	Non-switched memory CD19 + CD27 + sIgD+	509/48% ↑	32/39% N	41/38% N	43/7% N	44/48% N	0 ↓	30/24% N	54/22% N	7/7% ↓	0 ↓	3/15% ↓	13/11% N
	Switched-memory CD19 + CD27 + sIgD-	18/2% ↓	21/25% N	20/19% N	63/10% N	4/4% ↓	0 ↓	26/21% N	49/20% N	8/8% ↓	18/27% ↓	2/9% ↓	4/3% ↓
	T CD3+	1837/51% N	415/50% ↓	337/28% ↓	1256/44% N	622/57% ↓	264/77% ↓	283/37% ↓	621/28% ↓	174/18% ↓	855/62% N	325/62% ↓	450/52% ↓
	T CD4+	895/25% ↓	216/26% ↓	132/11% ↓	578/46% ↓	390/36% ↓	176/52% ↓	174/23% ↓	358/16% ↓	116/12% ↓	220/16% ↓	173/33% ↓	212/24% ↓
	T CD45RA+:CD45RO+	0.15 ↓	0.04 ↓	0.07 ↓	0.36 ↓	0.01 ↓	0.02 ↓	0.27 ↓	0.25 ↓	0.27 ↓	0.20 ↓	0.25 ↓	0.15 ↓
	Naïve Th CD4 + CD27 + CD45RA+	119/13% ↓	16/7% ↓	3/2% ↓	46/36% N	44/47% N	1/1% ↓	22/13% ↓	60/17% ↓	11/9% ↓	14/6% ↓	35/20% ↓	99/47% ↓
	RTE CD4 + CD31 + CD45RA+	61/7% ↓	16/7% ↓	1/0% ↓	437/59% N	10/2,5% ↓	2/1% ↓	12/7% ↓	19/5% ↓	28/24% ↓	18/8,3% ↓	25/14% ↓	77/36% N
	Central memory Th CD4 + CD27 + CD45RA-	650/73% ↑	177/82% N	108/81% N	180/24% N	332/85% N	71/45% ↓	126/72% N	277/77% N	100/86% ↓	154/70% ↓	111/64% ↓	101/48% ↓
	Effector memory Th CD4 + CD27-CD45RA-	129/14% ↑	22/10% N	17/13% N	52/7% N	43/11% N	103/58% N	26/15% N	20/6% N	5/4% N	47/21% N	21/12% N	9/4% ↓
	CXCR5+ Follicular Th CD4 + CD185 + CD45RO+	81/23% N	20/11% N	17/10% N	85/27% N	97/26% N	8/5% ↓	25/21% N	82/29% N	18/19% N	46/23% N	34/25% N	52/36% N
	T CD8+	1069/29% N	91/11% ↓	168/14% ↓	428/15% N	140/13% ↓	65/19% ↓	40/5% ↓	108/5% ↓	48/5% ↓	618/45% N	99/19% ↓	149/17% ↓
	Naïve Tc CD8 + CD197 + RA+	277/45% ↓	16/18% ↓	5/3% ↓	159/37% N	11/8% ↓	27/42% N	6/16% ↓	20/19% ↓	5/9% ↓	0 ↓	17/17% N	52/35% N
	Central memory Tc CD8 + CD27 + CD197 + CD45RA-	184/30% ↑	25/27% N	17/10% N	14/3% N	55/39% ↑	7/10% N	13/5% N	3/2% ↓	4/8% N	110/18% ↑	11/11% N	5/3% N
	Effector memory Tc CD8 + CD197-CD27-CD45RA-	15/2% N	12/13% ↓	41/25% ↓	31/7% ↓	5/3% ↓	7/10% ↓	2/6% ↓	6/6% ↓	2/5% ↓	358/58% N	39/39% ↓	6/4% ↓

### Infections

An in-depth analysis of individual patients' infectious history showed that all the patients studied suffered from respiratory tract infections yet the course of the lung and airway disease was variable. In two out of twelve patients, one with the HIGM A-T and another one with the non-HIGM A-T phenotypes, aged 17 and 18 years, respectively, an interstitial lung disease (ILD) with concomitant chronic obstructive airway disease was noted. While the tracheal aspirate cultures were positive in all the six HIGM A-T patients, in two out of six non-HIGM A-T children the cultures did not show bacterial pathogens. The most frequently cultured pathogen was *Streptococcus pneumoniae*, which was identified in five and two HIGM and non-HIGM A-T patients, respectively. It is worth noting that an opportunistic pathogen, *Pseudomonas aeruginosa* was identified in cultures exclusively from children with the HIGM A-T variant. Chronic infections with herpes viruses, such as *cytomegalovirus* (CMV), *Epstein-Barr virus* (EBV), and *human herpes virus 6* (HHV6) assessed as peripheral blood viral loads were found in four HIGM A-T patients and only two out of six non-HIGM A-T patients. The results of microbiologic investigations in all the A-T children studied and a comparison between HIG and non-HIGM patients are displayed in Table 2.

**Table 2:** The summary of infections and immune dysregulation disorders in HIGM A-T and non-HIGM A-T patients.

Phenotype	HIGM-AT						Non-HIGM-AT					
Patient No	Pt 1	Pt 2	Pt 3	Pt 4	Pt 5	Pt 6	Pt 7	Pt 8	Pt 9	Pt 10	Pt 11	Pt 12
Immune dysregulation		AIHA NHL	AIN	AIHA Chronic hepatitis		AIHA Chronic hepatitis Cutaneous and systemic granulomatosis			JIA			
Infections	Rhinovirus	Enterovirus HHV6	Rhinovirus	CMV EBV	CMV EBV	EBV HHV6		Bocavirus Rhinovirus		CMV EBV		
	Strep. pneumoniae H. influenzae	Staph. aureus H. influenzae P. aeruginosa	Strep. pneumoniae H. influenzae P. aeruginosa	Strep. pneumoniae H. parainfluenzae	Strep. pneumoniae H. parainfluenzae	Strep. pneumoniae Staph. aureus Serratia marcescens H. parainfluenzae P. aeruginosa		Strep. pneumoniae		Strep. pyogenes	H. parainfluenzae	Strep. pneumoniae
Outcome				Liver failure deceased		Liver failure deceased						

### Immune dysregulation

Immune dysregulation phenotypes were two-fold more frequently observable in HIGM A-T than in non-HIGM A-T patients. Autoimmune hemolytic anemia (AIHA) was the most common autoimmune disorder as it was seen in three out of six HIGM A-T individuals. Other autoimmune diseases such as autoimmune neutropenia (AIN), and juvenile idiopathic arthritis (JIA), were seen in single cases (the first occurred in HIGM A-T, whereas the latter was the only autoimmune disorder diagnosed in a non-HIGM A-T patient). Inflammatory chronic liver disease was the organ-specific immunopathology in two patients with the HIGM A-T variant who deceased due to liver failure. Non-Hodgkin B-cell lymphoma (NHL) was a sequela in one HIGM A-T patient. Two children with the HIGM A-T variant died, aged 6 and 17 years old, both due to chronic liver failure, pancytopenia, and chronic EBV infection. In one of these patients, chronic hepatitis was accompanied by cutaneous and systemic granulomatosis, involving the palate, pharynx, larynx, and lungs. The summary of immune dysregulation disorders with discrimination between HIGM and non-HIGM A-T patients is displayed in Table 2.

## Discussion

The combined immunodeficiency belongs to the complex and heterogeneous individual phenotype in ataxia telangiectasia patients. The impaired T cell compartment manifesting as thymic dysfunction in generation recent thymic emigrants and naive T CD4 + helper cells and also suppressor/ cytotoxic T CD8 + cells is a universal feature of A-T, observable in both HIGM and non-HIGM variants. A decrease in CD4 + and CD8 + naive, central memory, and terminally differentiated effector memory CD4+ T cells was shown in the peripheral T cell compartment in all the A-T affected individuals. This impaired generation of naive CD4+ T helper cells with a low number of RTE and a CD4 + CD45RA+: CD4 + CD45RO + ratio has, therefore, no discriminatory significance between our HIGM and non-HIGM A-T patients. Consequently, disturbed T cell neogenesis and reduced T cell repertoire diversity contribute to the increased predisposition to infections (28–30, 35). The pathogenesis of defective T cell homeostasis in A-T patients is complex and thymus dysfunction with low thymic output (36), disorders of the cell cycle checkpoints regulation, oxidative stress responses (37, 38), premature immune aging process (39, 40) as well as increased CD95-mediated apoptosis (41) have been postulated as candidate contributory pathways. Among B cell subsets, a reduced number of CD19 + CD27- cells representing naive B cells and corresponding with the impairment of the bone marrow output has been observed in our A-T children studied, consistently with other reports (42, 43). The switched-memory B cells have been depleted in three out of six HIGM and four out of six non-HIGM children, which is an unexpected finding in individuals with elevated serum IgM levels reflecting an impaired class switch recombination (CSR) (31–34) being the major criterion for stratifying our patients with A-T. We have also noted that IgA serum levels were admittedly higher in the non-HIGM group with normal or partially deficient IgA in as many as four patients. Referring to the recent report (44) on the predictive role of IgA as a simple, surrogate marker in anticipating the poorer prognosis in A-T patients, our five HIGM A-T presented with a complete IgA deficiency. The HIGM group also more frequently presented a deficit in IgG subclass generation and this parameter has also been shown to correlate with shorter survival in A-T (45). These findings mirror a deeply impaired process of immunoglobulin class switching and correspond with a higher risk of respiratory tract viral, most frequently due to *Rhinovirus*, and bacterial, such as *Streptococcus pneumoniae* and *Haemophilus influenzae* infections (46–48). Interestingly, the heterogeneity of IgG subclass distribution had an important clinical significance also in patients with non-HIGM A-T. Whereas the IgG2 deficiency was associated with recurrent infections due to *Streptococcus pneumoniae*, in patients with IgG3 deficiency no bacterial pathogens or *Haemophilus parainfluenzae* were cultured in the airways. Furthermore, the opportunistic pathogen, *Pseudomonas aeruginosa* which was cultured exclusively in patients with the HIGM A-T variant, is associated with a more severe course of lung disease and a higher risk of chronic respiratory failure (16, 49). In our group of HIGM A-T children, impaired CSR was associated with more severe clinical phenotypes, including autoimmune phenomena, granulomatous disorders, inflammatory organ-specific immunopathology, lymphoproliferation, and malignancy, consistently with previous reports (18, 24, 33, 34). Whereas in three out of all the six HIGM A-T patients, Epstein-Barr virus (EBV)-DNA was persistently identified in peripheral blood pointing to the EBV reactivation, important concerns are raised regarding the role of the dysfunctional ATM kinase activity in favoring the EBV life cycle and promoting lymphoproliferation and malignancy in immunocompromized A-T individuals (50, 51). Interestingly, our six-year-old A-T patient with HIGM variant, in whom non-Hodgkin lymphoma (NHL) was diagnosed, showed reactivation of human herpes virus (HHV)-6 but not EBV, which DNA was undetectable. This observation might be supported by studies of the immune response to HHV-6 in T-cell immunodeficient humans failing protection against the reactivation of the virus causing infectious, inflammatory, and malignant life-threatening complications (52, 53).

In conclusion, due to the rarity of A-T, our single-center group of pediatric patients is relatively small, a fact that presents as a major limitation of this study. Whereas we were able to stratify our A-T children into HIGM and non-HIGM groups according to the serum IgM levels, we observed B cell developmental impairment in both phenotypes. Accordingly, IgA deficiency was a universal feature in all but one A-T patient studied and did not play any discriminatory role between HIGM and non-HIGM groups. Likewise, low thymic RTE output and low naive T helper to memory T helper lymph cell ratio was the cardinal feature of both HIGM and non-HIGM A-T immunophenotypes. However, immune dysregulation associated with autoimmune disorders showed a striking predilection to the HIGM phenotype. Non-effective antibody maturation, expansion of IgM autoantibodies during immune response to infection, and an increased germinal center autoreactive B cell population have been proposed as contributing factors to autoimmune disorders in HIGM-AT (34). Furthermore, a fatality due to EBV expansion and liver failure was observable solely in HIGM A-T patients. It may therefore be assumed that more profound T cell dysfunction may be assigned to the defective immunoglobulin class switching and HIGM A-T phenotype with a poorer prognosis.

Our observations highlight the complexity of pathophysiology and symptomatology in A-T, a disease at the interface of immune deficiency, autoinflammation, organ-specific immunopathology, and malignancy with a more severe and unfavorable course associated with the HIGM A-T variant. Therefore, affected children require pediatricians' awareness, careful and attentive evaluation, and multidisciplinary care under the pediatric immunologist's supervision to improve prognosis.

## Data Availability

The raw data supporting the conclusions of this article will be made available by the authors, without undue reservation.
